# Customized Deep Eutectic Solvents as Green Extractants for Ultrasonic-Assisted Enhanced Extraction of Phenolic Antioxidants from Dogbane Leaf-Tea

**DOI:** 10.3390/foods10112527

**Published:** 2021-10-21

**Authors:** Ruimin Wang, Weimin Zhang, Ruiping He, Wu Li, Lu Wang

**Affiliations:** 1 School of Food Science and Engineering, Hainan University, Haikou 570228, China; wangruimin2020@163.com (R.W.); zhwm1979@163.com (W.Z.); he15337389982@163.com (R.H.); leewuu@163.com (W.L.); 2 Key Laboratory of Food Nutrition and Functional Food of Hainan Province, Hainan University, Haikou 570228, China; 3 Key Laboraory of Tropical Fruits and Vegetables Quality and Safety for State Market Regulation, Hainan University, Haikou 570228, China

**Keywords:** Dogbane leaf-tea, phenolic compounds, deep eutectic solvents, ultrasonic-assisted extraction, response surface methodology, antioxidant activity, anti-glucosidase activity

## Abstract

This study evaluates the application of eco-friendly deep eutectic solvents (DESs) in the extraction of phenolic antioxidants from dogbane leaf-tea (DLT). The results showed DESs with lower viscosity allowed an efficient extraction of significantly higher contents of total phenolics or flavonoids. An innovative and high-efficient solvent, choline chloride-levulinic acid (ChCl-LevA), was screened and used in ultrasonic-assisted extraction (UAE) of phenolic compounds from DLT. According to full factorial design experimental results, total phenolic content (TPC), total flavonoid content (TFC), antioxidant activity, and anti-*α*-glucosidase activity (*α*-GIA) of the DLT extracts were simultaneously optimized by response surface methodology. Sonication temperature and water content in ChCl-LevA were found to be the major factors affecting the TPC, TFC, antioxidant activity, and *α*-GIA of DLT extracts. Under the optimum parameters (water content in ChCl-LevA was 45%, sonication temperature was 50 °C, and extraction time was 30 min), the measured results for all the responses were obtained as follows: TPC-91.38 ± 7.20 mg GAE/g DW, TFC-84.12 ± 3.47 mg RE/g DW, ABTS^+^-492 ± 7.33 mmol TE/g DW, FRAP-6235 ± 121 μmol Fe(II)/g DW and *α*-GIA-230 ± 7.59 mmol AE/g DW, which were consistent with the predicted values. In addition, strongly significant positive correlations were observed between TPC/TFC and bio-activities of the DLT extracts. HPLC results indicated high contents of (-)-epigallocatechin (4272 ± 84.86 μg/g DW), catechin (5268 ± 24.53 μg/g DW), isoquercitrin (3500 ± 86.07 μg/g DW), kaempferol 3-*O*-rutinoside (3717 ± 97.71 μg/g DW), and protocatechuic acid (644 ± 1.65 μg/g DW) were observed in the DLT extracts. In contrast to other extraction methods, ChCl-LevA-based UAE yielded higher TPC, TFC, individual phenolic contents, stronger antioxidant activity, and *α*-GIA. Scanning electron microscope (SEM) analysis further confirmed that ChCl-LevA-based UAE enhanced the disruption of cell wall structure, thereby making more phenolic antioxidants released from DLT. In short, ChCl-LevA-based UAE was confirmed to be an innovative and high-efficient method for extraction of phenolic antioxidants from DLT. Dogbane leaves can be considered as a good tea source rich in natural antioxidants.

## 1. Introduction

Dogbane (*Apocynum ventem* L.), belonging to the family of the Apocynaceae, is widely distributed in Western Europe, Central Asia, North America, and Northwestern of China [1,2]. In China, fresh leaves of *A. ventem* L. have long been used as an ingredient for tea product (namely dogbane leaf-tea) owing to its multiple pharmacological activities [3,4]. Many researchers have confirmed that dogbane leaf-tea (DLT) extracts contain various health-related bio-active components, such as phenolics, coumarins, amino acids, fatty acid, and polysaccharides, etc. [1,2]. Among them, phenolics are the major active constituents of DLT extracts, which have anti-oxidant, anti-hypoglycemic, anti-hypotensive, anti-depressant, blood lipid regulation, and liver protection effects [5]. Considering their benefits to human health, phenolic compounds are usually used as natural additives in food industry [6,7,8].

Normally, polar or non-polar active compounds from natural products are extracted by using organic solvents such as alcohols, ethyl acetate, chloroform, and acetone, etc. [9,10]. However, traditional organic solvents are usually flammable, volatile, and toxic in different degrees [11,12]. Legislation of European Union has pointed out that the top priority currently is to gradually decrease the use of volatile organic solvents from 2010 to 2050. Additionally, considering the close relationship between diet phenolics and health, the requirements of foods formulation with active components from medicine and food homology are increasingly higher. From these perspectives, it is urgent to develop effective and green technologies for extracting bio-active components from natural plants. Deep eutectic solvents (DESs), as a type of novel eco-friendly solvents, have low melting points and are normally synthesized by heating the mixtures of two or more eco-friendly components with relatively high melting points [13,14]. Owing to their advantages of low costs, biodegradability, eco-friendliness, and non-toxicity, DESs have been increasingly applied in the extraction of bio-active compounds including phenolics, alkaloids, and saponins [15,16,17]. Currently, ultrasonic technique has been introduced into active compounds extraction because of its lower cost, high-efficiency, eco-friendliness, and easiness in scaling up [18,19]. On one hand, the cavitation effect caused by ultrasonication can rupture the cell wall structure, making more active compounds released from plant materials. On the other hand, ultrasonic process can enhance the energy and mass transfer of extractives in solvent system by declining the diffusion boundary layers. Hence, the ultrasonic-assisted extraction (UAE) process using eco-friendly DESs is expected to enhance the extraction of active compounds from natural products [18,19,20]. However, there have been no reports on enhancing the extraction of phenolic antioxidants from DLT by using eco-friendly DESs-based UAE technique.

In this study, a high-efficiency and green solvent was firstly screened out amongst a series of solvents and used in the extraction if the phenolic compounds from DLT, and the solvents characterization were analyzed for the first time. Afterwards, the parameters optimization of the screened DES-based UAE process was carried out. Finally, chemical compositions and biological activities of the DLT extracts extracted with different methods were investigated. The aim of this study is to exploit a novel, effective, and green method for enhanced extraction of phenolic compounds from DLT.

## 2. Materials and Methods

### 2.1. Plant Materials and Chemicals

Dogbane leaf-tea (DLT) was provided by Great Northwest Pharmaceutical Co., Ltd. (Bozhou, Anhui, China). The freeze-dried leaves were ground into powder, sieved with 60 mesh screens to obtain particles <0.3 mm, and stored at 4 °C. Phenolic standards (HPLC grade, >99.7%), Folin-Ciocalteu’s reagent, Trolox, 2,2′-azino-bis (3-ethylbenzothiazoline-6-sulfonic acid) diammonium salt (ABTS), 2,4,6-tripyridyl-*s*-triazine, 4-*N*-trophenyl-*α*-*D*-glucopyranoside (*p*-NPG), and *α*-glucosidase (CAS Number: 9001-42-7) were purchased from Sigma-Aldrich Chemical Co. (Steinheim, Germany). Mobile phases for HPLC analysis were purchased from Fisher Scientific (Waltham, MA, USA). Analytical grade-chemicals were purchased from Aladdin (Shanghai, China).

### 2.2. Preparation and Chemical Characteristics Analysis of DESs

As shown in Table 1, DESs were prepared by heating the mixture of multiple components to 80 °C under magnetic stirring until the formation of homogeneous transparent liquid [2]. The pH value of the prepared DESs was measured by an electronic handheld Model PHSJ-6L pH meter (Shanghai Leici instruments Factory, Shanghai, China). Adding 30% water in the prepared DESs was conducive to reducing the viscosity of DESs. The viscosity of the different types of DESs was determined using a HAAKE MARS 40 type rheometer (ThermoFisher Scientific, Karlsruhe, Germany). The viscosity of each DES was determined in triplicate, in order to obtain the averaged value. Fourier transform infrared spectra (FTIR) of extraction solvents were measured by using a Nicolet iS50 FTIR spectrometer. The FTIR spectra were analyzed at 4 cm^−1^ spectral resolution, 32 scans over 400–4000 cm^−1^ range, with a Smart Omni accessory.

### 2.3. Total Phenolic Content (TPC) and Total Flavonoid Content (TFC)

TPC in the DLT extracts was determined using Folin-Ciocalteu method [21]. The calibration curve of gallic acid as a standard (Y = 0.0029X + 0.0241, *R*^2^ = 0.9989) was drawn. TPC was expressed as mg gallic acid equivalent (GAE)/g dry weight (DW). TFC was measured by the aluminum chloride colorimetric method proposed by Wang et al. [22]. The absorbance was read at 510 nm and compared to a rutin calibration curve (Y = 0.0005X − 0.0007, *R*^2^ = 0.9978). TFC was expressed as mg rutin equivalent (RE)/g DW.

### 2.4. Antioxidant Activity In Vitro Assays

ABTS^+•^ scavenging activity and FRAP assays were conducted to evaluate antioxidant activities in vitro of the DLT extracts. The ABTS^+•^ scavenging activity of the DLT extracts was determined according to the method proposed by Re et al. [23]. The result of ABTS^+•^ scavenging activity assay was expressed as millimoles of Trolox equivalents (TE)/g DW. The FRAP of the DLT extracts was measured by the method proposed by Benzie et al. [24]. The FRAP value was expressed as μmol Fe^2+^ equivalents per gram sample in dry weight (Fe(II)E)/g DW.

### 2.5. α-Glucosidase Inhibitory Activity (α-GIA) Assay

The *α*-glucosidase inhibitory activity was measured using the method of Cai et al. [25]. Briefly, 100 μL of diluted DLT extracts or different concentrations of acarbose were mixed with 50 μL of 0.5 U/mL *α*-glucosidase solution and then incubated at 37 °C for 10 min. After that, 100 μL of substrate *p*-NPG solution (5 mM) were added and incubated for another 20 min. Lastly, 500 μL of Na_2_CO_3_ solution (1 M) was added to terminate the reaction, and the absorbance of reaction solution was measured at 405 nm. The inhibitory activity of α-glucosidase was expressed as millimoles of acarbose equivalents (AE) per gram sample in dry weight (mmol AE/g DW).

### 2.6. Experimental Designs

#### 2.6.1. Full Factorial Design Experiments

Full factorial design (FFD) experiments designed by Design Expert 10.0.0 software (Sta-Ease, Minneapolis, MN, USA) were conducted for initial screening of key UAE parameters [13,26]. A five-factor two-level FFD experimental was carried out to investigate the influences of water content in DES (A), liquid to solid ratio (B), extraction time (C), ultrasonication power (D), and ultrasonication temperature (E) on TPC and TFC (Table S1). The key UAE parameters exhibiting significant effects on the responses were illustrated by the Pareto chart of the standardized effects (Figure S1).

#### 2.6.2. Response Surface Methodology (RSM)

Based on the results of above FFD experiments, the important influencing factors of the responses variables were further optimized by RSM-based central composite design (CCD). A total of 20 experimental runs in a randomized order were determined (Table S2 and Table 2). Water content in DES (*X*_1_), extraction time (*X*_2_), and ultrasonic temperature (*X*_3_) were selected as the key independent variables. Response variables including TPC, TFC, ABTS^+•^, FRAP, and *α*-GIA were investigated. The predictive equations of RSM were used to analyze the regression equations, response surfaces, contour plots, and determine the optimal values of the responses. The second-order response function for RSM was expressed by the equation below (Equation (1)).

(1) $$Y=\lambda_{0}+\overset{k}{\underset{i=1}{\sum }}\lambda_{i}X_{i}+\overset{k=1}{\underset{\begin{array}{c} i=1\\ j>i \end{array}}{\sum }}\overset{k}{\underset{j=2}{\sum }}\lambda_{ij}X_{i}X_{j}+\overset{k}{\underset{i=1}{\sum }}\lambda_{ii}X_{i}^{2}$$

where *λ*_0_, *λ_i_*, *λ_ii_*, and *λ_ij_* represent the regression coefficients of intercept, linear, quadric, and interaction, respectively; *X_i_* and *X_j_* represent the independent variables; *Y* represents the responses variables (TPC, TFC, ABTS^+•^, FRAP, and *α*-GIA); *k* is number of variables.

### 2.7. Comparison of ChCl-LevA Based-UAE and Other Methods

#### 2.7.1. Heating Extraction (HE)

First, 0.5 g of DLT powder was mixed with 10 mL of water, MeOH, or ChCl-LevA (45% water content) at a liquid-solid ratio of 20:1 (mL/g), respectively. The heating extraction procedure was performed in a XMTD-204 thermostat water bath (Shanghai, China) at 50 °C for 30 min, and then centrifugal treatment (8000× *g*, 5 min) was carried out to collect the supernatant.

#### 2.7.2. Microwave-Assisted Extraction (MAE)

For this procedure, 0.5 g of DLT powder and 10 mL of ChCl-LevA (45% water content) were mixed evenly at a liquid-solid ratio of 20:1 (mL/g), and the extraction procedure was performed in a NN-GF37JW microwave oven (Osaka, Japan) at 400 W for 30 s before collecting the supernatant by centrifugation at 8000× *g* for 5 min.

#### 2.7.3. Ultrasound-Assisted Extraction (UAE)

Ultrasound-assisted extraction procedure was performed by mixing 0.5 g of DLT powder with 10 mL of ChCl-LevA (45% water content) at a liquid-solid ratio of 20:1 (mL/g) in an ultrasonic water bath at 50 °C for 30 min (KQ-400KDE, Kunshan, China). The supernatant was collected by centrifugation at 8000× *g* for 5 min for subsequent analysis.

### 2.8. HPLC-DAD Analysis

Phenolic compositions in the DLT extracts were quantified by using an Agilent 1260 HPLC-DAD system coupled with a DAD and a Waters SunFire C_18_ column (Waters, 250 mm × 4.6 mm, 5 μm, Milford, CA, USA) [20]. Mobile phases consisted of acetonitrile (A) and 0.1% formic acid–water (B), and the gradient elution program was set as follows: 0–5 min, 15% B; 5–25 min, 25–35% B; 25–40 min, 25–50% B; 40–45 min, 85% B; and 45–50 min, 15% B. The column temperature was 30 °C, flow rate was 0.8 mL/min, the injection volume was 10 μL, and the detection wavelength was carried out at 280 nm [19,20]. The contents of the identified phenolic compounds were expressed as μg/g DLT in dry weight.

### 2.9. Scanning Electron Microscopy (SEM) Analysis

Microscopic morphology of the samples before and after extraction was analyzed by using a Verious G4 UC scanning electron microscope. After the vacuum freeze-drying treatments, the raw and extraction residues were placed on conductive glue and plated gold, and then photographed at an operating voltage of 2.0 kV.

### 2.10. Statistical Analysis

The data were expressed as means ± standard deviations. FFD, CCD and regression coefficient analysis were performed using Design Expert software version 10.0 (Stat-Ease Inc., Minneapolis, MN, USA). All data were analyzed by one-way ANOVA, post-hoc Tukey’s test and processed in the IBM SPSS Statistics software. The difference was considered significant when *p* < 0.05.

## 3. Results and Discussion

### 3.1. Screening and Physical-Chemical Properties of DESs

**Figure 1:**
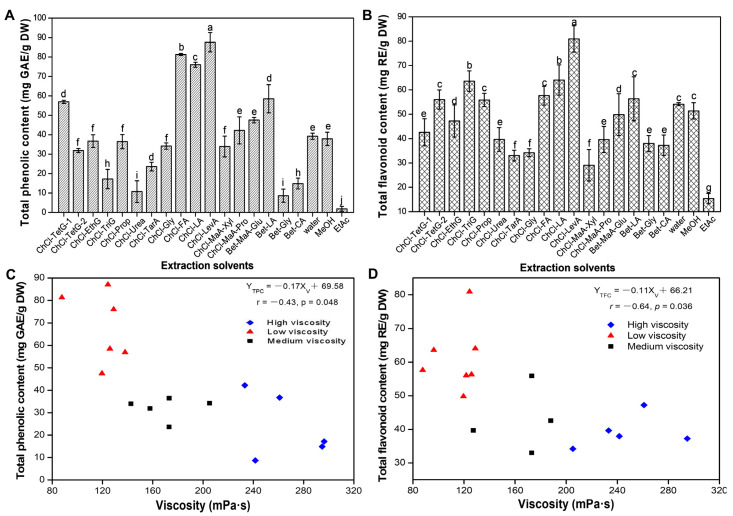
Total phenolic content (TPC, **A**), total flavonoid content (TFC, **B**) of dogbane leaf-tea (DLT) extracts extracted with various solvents; TPC or TFC of the DLT extracts versus viscosity of 17 types of DESs (**C**,**D**). Different lowercase letters indicate significant differences.

The extraction efficiencies of 17 kinds of DESs including acidic-based DESs, amide-based DESs, and sugar-based DESs were evaluated according to the yielded TPC and TFC. Figure 1A,B shows the TPC and TFC of the DLT extracts extracted by DESs and conventional solvents (water, methanol, and EtAc). It is clear that ChCl-LevA led to the highest extraction yield of TPC (87.61 mg GAE/g DW) and TFC (80.91 mg RE/g DW). In addition, ChCl-FA and ChCl-LA also showed excellent extraction efficiency for TPC and TFC. The water and methanol had similar extraction efficiency. Compared with methanol, ChCl-LevA brought 2.30-times and 1.58-time higher TPC and TFC, respectively. EtAc showed the worst extraction ability for phenolic compounds, which is in line with our previous study [13,27]. Additionally, it can be seen that TPC and TFC in acidic-based DESs (ChCl-FA, ChCl-LA and ChCl-LevA) extracts were significantly higher than those in other DESs extracts, which is consistent with the viewpoint reported by Wu et al. [13].

Some researchers have confirmed that the viscosity, pH, and polarity of DESs have great influences on the extraction of bio-active compounds [28]. In this work, the pH and viscosity values of the prepared DESs (adding 30% water) are shown in Table 1. Five types of DESs (ChCl-TarA, ChCl-FA, ChCl-LA, ChCl-LevA, and ChCl-MaA-Xyl) have relatively lower pH value than other DESs. ChCl-TarA and ChCl-MaA-Xyl with low pH value did not show high extraction efficiency for TPC and TFC. Three DESs (ChCl-FA, ChCl-LA, and ChCl-LevA) with low pH and viscosity values showed excellent extraction efficiency for TPC and TFC. Therefore, the pH value of DESs may not be the only factor affecting the extraction efficiency, which is consistent with the results of Wu et al. [13]. Generally, the viscosity of solvent is also an important factor affecting the cavitation effect and mass-/energy-transfer during sonication extraction [29,30]. It can be observed that the viscosity of the prepared DESs ranged from 87.63 mPa·s to 294.81 mPa·s. The relationships between the viscosity of DESs and the extraction yield of TPC or TFC are shown in Figure 1C,D. Based on the extraction efficiency of TPC and TFC, 17 types of DESs were divided into three categories: high viscosity (>210 mPa·s), medium (140–210 mPa·s), and low viscosity (<140 mPa·s). It can be observed that DESs with lower viscosity values indicated significantly higher extraction yields for total phenolics and total flavonoids, which was in agreement with the results of Fu et al. [31]. In a short, the varying physical and chemical properties (types, pH, viscosity, and polarity) of DESs reflect the affinities between the solvents and extract compounds, and thereby affect the extraction efficiency for phenolic compounds [32,33,34]. In the present work, ChCl-LevA was adopted as the best suitable solvent.

### 3.2. FT-IR Spectra of Extraction Solvents

**Figure 2:**
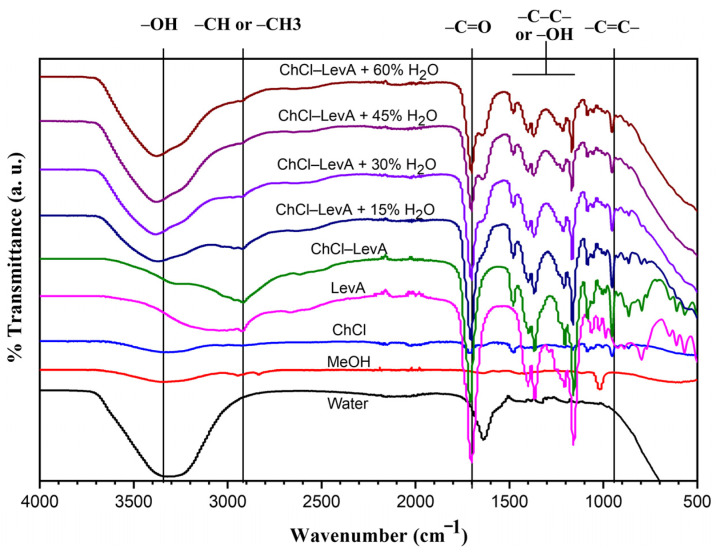
FT-IR spectra of water, MeOH, ChCl, LevA, ChCl-LevA (1:2) and ChCl-LevA with different addition amounts of water in solvent (15, 30, 45, and 60% *w*/*w*).

Figure 2 shows FTIR spectra of water, MeOH, DES components (choline chloride and levulinic acid), as well as ChCl-LevA without and with addition of water (15, 30, 45, and 60%). Normally, FTIR spectra can reflect the chemical bond structures of the extraction solvents. As expected, the differences in vibrational bands and bandwidths were observed in different solvents. Water, MeOH, ChCl-LevA, and ChCl-LevA with addition of water exhibited similar vibrational bands around 3100 cm^−^^1^, 3600 cm^−^^1^, and 1620 cm^−^^1^ (corresponding to -OH or H-O-H stretching vibration), which was due to hydrogen bonding on solvents molecules. For ChCl-LevA, with the increase of water content, it can be found that the intensity of the peak band at 3100–3600 cm^−^^1^ (corresponding to hydrogen bonds) was evidently strengthened, while the intensity of FTIR peak bands at 2800–3000 cm^−^^1^ and 1705 cm^−^^1^ (corresponding to -C=O bond stretching vibration) was gradually weakened. The results demonstrated that hydrogen bonds between water and DES components were generated during ChCl-LevA formation. Several peaks at 1450–1100 cm^−^^1^ were due to -CH stretching or -OH deformation. Peaks at 1185 cm^−^^1^ were due to -C-C- stretch or -C-O-C- stretching vibration. Some researchers have verified that the presence of hydrogen bonds can decrease the melting temperature and viscosity of DES system [35]. In addition, an appropriate water content in DES can enhance mass-/energy- transfer and thereby improve the extraction efficiency of phenolic compounds. However, increasing the water content in DES did not always enhance their extraction efficiency, which may be due to the fact that high contents of water in DES can weaken hydrogen bonds among DESs components, thus altering their viscosity, polarity, and pH [33,36]. Herein, no information focused on how the addition of water content in ChCl-LevA either weakens the formation of hydrogen bonds or affects its extraction efficiency for phenolic compounds.

### 3.3. Modeling of UAE Process Conditions

Full factorial design (FFD) experiments were carried out to evaluate the effect degree of the influencing factors including water content in DES (A), liquid-solid ratio (L/S, B), extraction time (C), sonication power (D), and sonication temperature (E) on the responses TPC and TFC (Table S1). It can be found that the independent variables A, C, and E were significantly correlated with TPC (*p* < 0.05), but the variables D and E were not significantly correlated with TPC (*p* > 0.05) (Figure S1A). From Figure S1B, the independent variables A and C were found to be significantly correlated with TFC (*p* < 0.05), while the interactive factor of extraction time and sonication temperature (CE) had a statistically significant effect on TFC. According to the results of FFD experiments, factors including water content in DES, extraction time, and sonication temperature were selected for further optimization of TPC, TFC, ABTS^+•^, FRAP, and α-GIA through RSM-based CCD.

Table S2 and Table 2 show the matrix of experimental design, the predicted values, and the measured values. It can be observed that the measured values of TPC, TFC, ABTS^+•^, FRAP, and α-GIA ranged from 32.38−94.79 mg GAE/g DW, 28.81−86.95 mg RE/g DW, 222−495 mmol TE/g DW, 3280−6348 μM Fe(II)E/g DW, and 61.50−220 mmol AE/g DW, indicating the necessity of optimization of UAE conditions. The measured values were consistent with the predicted values. In addition, extremely significant positive correlations were found between TPC and TFC yields and bio-activities of the DLT extracts: TPC vs. TFC (r = 0.909, *p* < 0.001), TPC vs. ABTS^+•^ (r = 0.793, *p* < 0.001), TPC vs. FRAP (r = 0.687, *p* = 0.001), TPC vs. α-GIA (r = 0.686, *p* = 0.001), TFC vs. ABTS^+•^ (r = 0.775, *p* < 0.001), TPC vs. FRAP (r = 0.735, *p* = 0.001), and TFC vs. α-GIA (r = 0.650, *p* = 0.002). The second-order polynomial equations of TPC, TFC, ABTS^+•^, FRAP, and α-GIA are as follows (Equations (2)–(6)):

(2) $$Y_{TPC}=83.23+16.15X_{1}+14.80X_{2}+3.21X_{3}+4.85X_{1}X_{2}+6.94X_{1}X_{3}-6.65X_{2}X_{3}-5.65X_{1}^{2}-9.66X_{2}^{2}-10.73X_{3}^{2}$$

(3) $$Y_{TPC}=72.79+9.51X_{1}+16.89X_{2}+4.99X_{3}+7.47X_{1}X_{2}+3.77X_{1}X_{3}-4.40X_{2}X_{3}-9.81X_{1}^{2}-5.64X_{2}^{2}-7.48X_{3}^{2}$$

(4) $$Y_{ABTS}=463.45+50.22X_{1}+27.45X_{2}-5.53X_{3}+26.79X_{1}X_{2}+33.18X_{1}X_{3}-14.24X_{2}X_{3}-41.30X_{1}^{2}-23.92X_{2}^{2}-0.28X_{3}^{2}$$

(5) $$Y_{FRAP}=584263+794.7X_{1}+51.40X_{2}+435.09X_{3}+239.72X_{1}X_{2}+109.06X_{1}X_{3}+18.59X_{2}X_{3}-435.60X_{1}^{2}-57.50X_{2}^{2}-193.25X_{3}^{2}$$

(6) $$Y_{\alpha -GIA}=151.08+46.51X_{1}+19.04X_{2}+10.20X_{3}-4.31X_{1}X_{2}+1.55X_{1}X_{3}+11.15X_{2}X_{3}-3.74X_{1}^{2}-21.56X_{2}^{2}-2.63X_{3}^{2}$$

The ANOVA results of the responses are shown in Table 3. It can be observed that the models well predicted the actual results of the responses. In general, high F-value implied a high significance of the model term. In this study, high F-values (F_TPC_ = 17.06, F_TFC_ = 16.18, F_ABTS_ = 6.91, F_FRAP_ = 30.10 and F_α-GIA_ = 16.21) and low *p*-values (<0.001) revealed that the RSM model is capable of predicting and optimizing the UAE procedure. In addition, high *R*^2^ values (*R*^2^_TPC_ = 0.9389, *R*^2^_TFC_ = 0.9701, *R*^2^_ABTS_ = 0.9296, *R*^2^_FRAP_ = 0.9849, and *R*^2^_α-GIA_ = 0.9507) and *R*^2^_Adj_ (*R*_Adj_^2^_TPC_ = 0.8838, *R*_Adj_^2^_TFC_ = 0.9054, *R*_Adj_^2^_ABTS_ = 0.8778, *R*_Adj_^2^_FRAP_ = 0.9522, and *R*_Adj_^2^_α-GIA_ = 0.9438) showed that there was a high consistency between predicted and measured values. The high adequacy precision (˃4.0) implied an adequate result of signal/noise, indicating high precision and reliability of the RSM mode [19,37]. In this study, high adequacy precision (AP) of 11.657, 10.900, 9.321, 20.811, and 10.304 was observed for TPC, TFC, ABTS^+•^, FRAP, and α-GIA, respectively. As shown in Table 3, it can be found that the *X*_1_ and *X*_2_ had significant linear correlations with TPC, *X*_1_^2^, *X*_2_^2^, and *X*_3_^2^ had significant quadratic relations with TPC, the interaction of *X*_1 × 3_ and *X*_2 × 3_ had a significant correlation with TPC (*p* < 0.05), but *X*_1 × 2_ had a non-significant correlation with TPC. *X*_1_, *X*_2_, and *X*_3_ had significant linear relations with TFC, *X*_1_^2^, *X*_2_^2^, and *X*_3_^2^ had significant quadratic relations with TFC. The interaction of *X*_1_ and *X*_2_ showed a significant relation with TFC (*p* < 0.05). ABTS^+•^ was mainly influenced by *X*_1_, *X*_2_, *X*_1 × 3_, *X*_1_^2^, and *X*_2_^2^. *X*_1_ had an extremely significant linear correlation with ABTS^+•^ (*p* < 0.001), and *X*_1_^2^ and *X*_2_^2^ had significant quadratic relations with ABTS^+•^ (*p* < 0.05). In addition, the interaction of *X*_1_ and *X*_3_ had a significant effect on ABTS^+•^. FRAP was mainly affected by *X*_1_, *X*_3_, *X*_1 × 2_, *X*_1_^2^, and *X*_3_^2^. *X*_1_, *X*_3_, *X*_1_^2^, and *X*_3_^2^ had significant linear and quadratic effects on FRAP, while *X*_2_ and *X*_2_^2^ had no significant effects on FRAP (*p* < 0.05). In addition, a significant interaction effect was observed between *X*_1_ and *X*_3_. With respect to α-GIA, *X*_1_, *X*_2_, and *X*_3_ had highly significant linear effects on α-GIA (*p* < 0.01), *X*_2_^2^ had an extremely significant quadratic effect on α-GIA (*p* < 0.001). The interaction of *X*_2_ and *X*_3_ had a significant influence on α-GIA (*p* < 0.05), while *X*_1 × 2_ and *X*_1 × 3_ had no significant effects on α-GIA.

**Figure 3:**
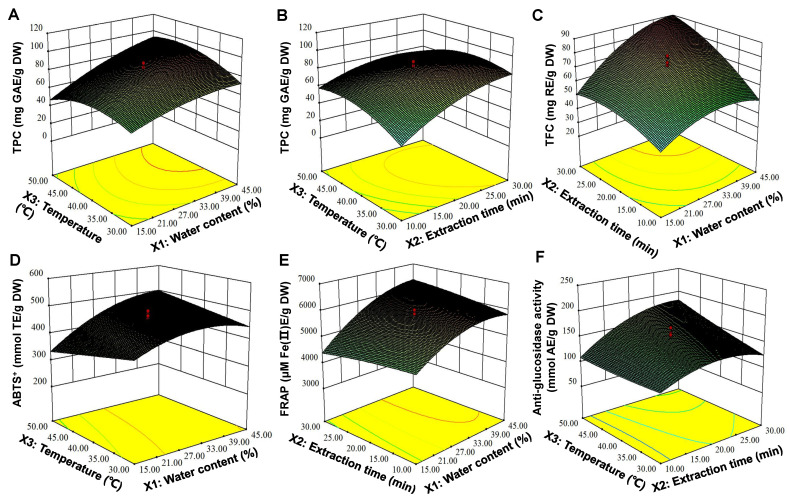
The 3D surface plots of interaction effects between the independent variables on TPC (**A**,**B**), TFC (**C**), ABTS^+•^ (**D**), FRAP (**E**), and α-GIA (**F**) of the DLT extracts.

According to Equations (2)–(6), it can be observed that *X*_1_, *X*_2_, and *X*_3_ had positive influences on all responses. The interaction of *X*1 and *X*_3_ had a positive effect on all responses. In addition, *X*_1_^2^, *X*_2_^2^ and *X*_3_^2^ exhibited negative quadratic effects on all responses. In order to provide an intuitive visualization of the interactions of the independent variables on the responses, 3D response surface plots and contour plots for RSM were plotted, as shown in Figure 3A−F and Figure S2A−F, respectively. It is clear that *X*_1_ was an important variable affecting the extraction of TP and TF from DLT. The 3D surface plots show that TPC, TFC, ABTS^+•^, and FRAP of the DLT extracts increased gradually with the increase of *X*_1_, and the maximum responses variables were obtained when *X*_1_ was set to 45% (Figure 3A,C,D,E). Wang et al. [19] also verified that *X*_1_ greatly influenced the viscosity and mass and energy transfer of extraction systems. An appropriate water content in DES can evidently enhance the extraction of phenolic compounds from DLT. In addition, TFC, FRAP, and α-GIA were decreased with the increase of *X*_2_ (Figure 3C,E,F). The interaction of *X*_2_ and *X*_3_ had an evident negative effect on TPC and α-GIA (Figure 3B,F), which is consistent with the results of Wang et al. [19].

### 3.4. Validation of the Optimal UAE Conditions

Based on the analysis of Design-Expert software, the optimal results for independent variables were obtained under water content of 45%, sonication temperature of 50 °C, and extraction time of 30 min; TPC (96.21 mg GAE/g DW), TFC (86.58 mg RE/g DW), ABTS^+•^ (481 mmol TE/g DW), FRAP (6473 μmol Fe(II)E/g DW), and *α*-GIA (218 mmol AE/g DW) were maximum predicted responses. Under the optimal conditions, the measured results of TPC, TFC, ABTS^+•^, FRAP and *α*-GIA were 91.38 ± 7.20 mg GAE/g DW, 84.12 ± 3.47 mg RE/g DW, 492 ± 7.33 mmol TE/g DW, 6235 ± 121 μmol Fe(II)/g DW and 230 ± 7.59 mmol AE/g DW, respectively (Table 3). There was a high consistency between measured and predicted results, and the relative low error values were low (2.12–5.47%), which indicates the RSM mode was suitable for the optimization of ChCl-LevA-based UAE.

### 3.5. Comparison of ChCl-LevA-Based UAE and Other Methods

**Figure 4:**
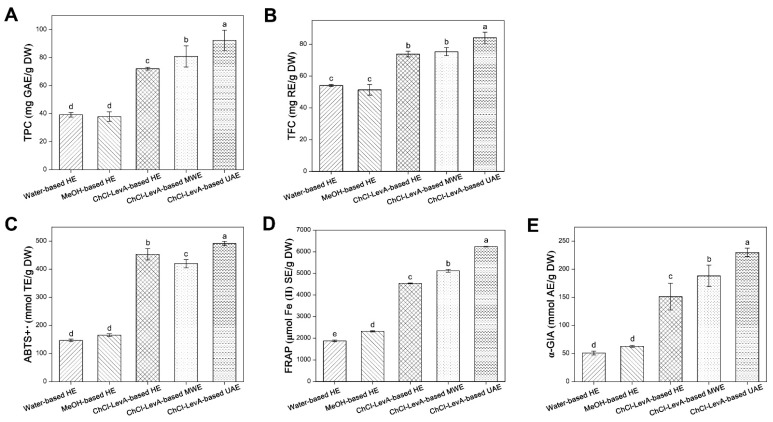
(**A**) TPC, (**B**) TFC, (**C**) ABTS^+•^ scavenging activity, (**D**) FRAP assays, (**E**) *α*-glucosidase (*α*-GIA) inhibitory activity in the DLT extracts extracted by different methods, where different lowercase letters indicate significant differences.

The extraction efficiencies of ChCl-LevA-based UAE and other methods were comparatively analyzed. As shown in Figure 4, it can be observed that ChCl-LevA-based UAE extract showed the highest TPC, TFC, ABTS^+•^, FRAP, and *α*-GIA under optimal conditions. For extraction processes using water and methanol as solvents, no significant differences were observed for all responses, except for FRAP. ChCl-LevA-based HE extracts exhibited higher TPC, TFC, ABTS^+•^, FRAP, and *α*-GIA values than water-based HE extracts and MeOH-based HE extracts. By contrast, ChCl-LevA-based MWE extracts indicated slightly higher TPC, TFC, FRAP, and *α*-GIA values than water-based HE extracts, MeOH-based HE extracts, and ChCl-LevA-based HE extracts, but lower TPC, TFC, ABTS^+•^, FRAP, and *α*-GIA values than ChCl-LevA-based UAE extracts. The results implied that sonication-synergistic ChCl-LevA can be considered as an eco-friendly and efficient solvent for extracting phenolic compounds from DLT.

**Figure 5:**
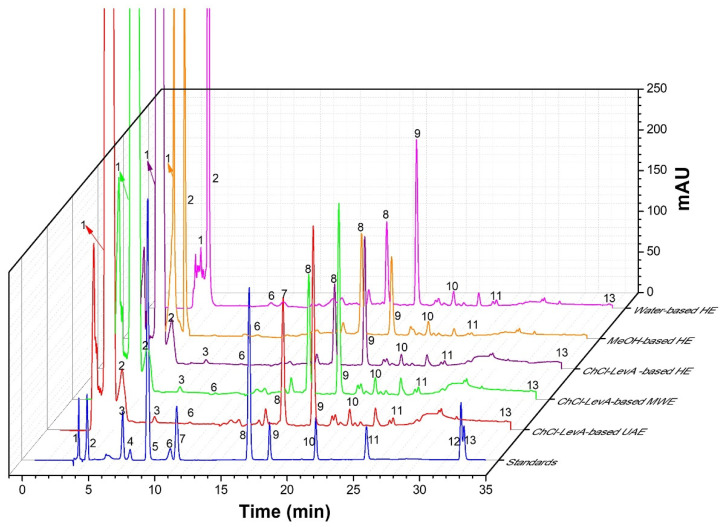
HPLC chromatograms of the DLT extracts obtained with different methods and the standards. Water-based HE, heating extraction with water; MeOH-based HE, heating extraction with MeOH; ChCl-LevA-based HE, heating extraction with ChCl-LevA; ChCl-LevA-based MWE, microwave extraction with ChCl-LevA; ChCl-LevA-based UAE, ultrasonic-assisted extraction with ChCl-LevA.

Chemical compositions in the DLT extracts were identified by comparing the retention time with the standards and referring to the previous publications [1,2]. Figure 5 shows the HPLC chromatograms of the DLT extracts obtained using ChCl-LevA-based UAE and other methods. Ten main compounds were quantified using internal standards by HPLC-DAD method, as shown in Table 4. It can be seen that both solvent type and extraction method greatly affected the contents of phenolic compounds extracted. Vanillic acid was only detected in water-based HE extract, while catechin was only detected in the ChCl-LevA extracts. High contents of (-)-epigallocatechin, isoquercitrin, and kaempferol 3-*O*-rutinoside were observed in all extracts. In contrast to heat extraction using normal solvents, ChCl-LevA extracts had higher contents of gallic acid, (-)-epigallocatechin, protocatechuic acid, rutin, isoquercitrin, kaempferol 3-*O*-rutinoside, and phloretin than water and MeOH extracts. Moreover, microwave or sonication-synergistic ChCl-LevA evidently enhanced the extraction of phenolic compounds from DLT. In addition, ChCl-MaA-based UAE extracts had higher contents of (-)-epigallocatechin (4272 ± 84.86 μg/g DW), catechin (5268 ± 24.53 μg/g DW), protocatechuic acid (644 ± 1.65 μg/g DW), isoquercitrin (3500 ± 86.07 μg/g DW) and kaempferol 3-*O*-rutinoside (3717 ± 97.71 μg/g DW) than ChCl-MaA-based MWE extracts and ChCl-LevA-based HE extracts, and there were no significant differences in the contents of rutin, myricetin and phloretin between ChCl-MaA-based UAE extracts and ChCl-MaA-based MWE extracts.

Recently, some innovative extraction techniques with tailor-made natural DESs as solvents have been widely used for high-efficient extraction of phenolic compounds from natural products [38,39]. Wang et al. [19] studied an ultrasonic method with novel ChCl-based DES (formed with choline chloride and malic acid), and found that it showed excellent efficiency in extracting natural antioxidants from partridge leaf-tea. Chanioti and Tzia [40] adopted modern innovative techniques (ultrasound, microwave, homogenization, and high pressure) with ChCl-based DESs as solvents to extract phenolic compounds from olive pomace, and found that homogenization-assisted extraction combined with choline chloride: citric acid (1: 2) yielded the highest TPC. In this study, it was found that ChCl-LevA-based UAE exhibited a better performance in extracting the phenolic compounds from DLT than other extraction methods, which is in line with the result of Fu et al. [31], who reported that sonication-synergistic deep eutectic solvent strengthened the destruction of the cell wall structure of *Carya cathayensis* peel, thereby allowing greater release of bio-active compounds.

**Figure 6:**
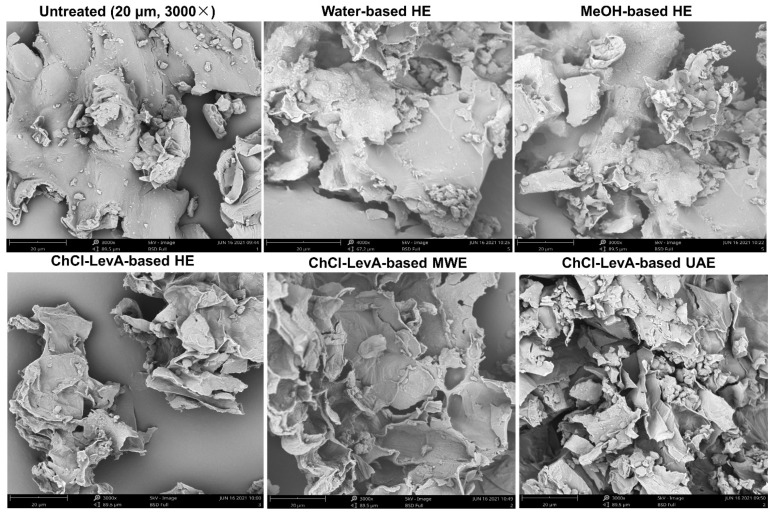
Scanning electron microscopy (SEM) analysis of the raw DLT and the DLT extraction residues after extraction with different methods. Water-based HE, heating extraction with water; MeOH-based HE, heating extraction with MeOH; ChCl-LevA-based HE, heating extraction with ChCl-LevA; ChCl-LevA-based MWE, microwave extraction with ChCl-LevA; ChCl-LevA-based UAE, ultrasonic-assisted extraction with ChCl-LevA.

The effects of different extraction methods on microscopic morphology of cell wall surfaces were also investigated, as shown in Figure 6. It can be observed that different extraction methods/solvents led to evident microscopic morphology changes of cell wall structures as compared with untreated DLT. Surfaces morphology of untreated samples was relatively massive, thick and opaque. There was no significant difference in external surface morphology of the samples treated with water and EtOH. However, the external surfaces of the samples treated with ChCl-LevA displayed more cracks and chasms, and became thinner and more transparent. In particular, the samples treated by ChCl-LevA combined with ultrasonic or microwave exhibited dramatically ruptured surfaces, with more chasms and pores. Usually, secondary metabolites exist in vacuole structure of plant matrix, which destroy cells structures, making more metabolites released from plant matrix [31,41,42]. In this study, ultrasonic extraction process using ChCl-LevA as solvent strengthened the disruption of the cell structures, thereby improving extraction of phenolic compounds from dogbane leaf-tea, which may be due to erosion capacity and high penetration of DESs towards plant cell walls [31]. In addition, the cavitation effects (namely transient high shear force) driven by ultrasonic or microwave also intensified the disruption and loosening of plant matrix [19,31,43].

## 4. Conclusions

In this work, ChCl-LevA as a high-efficiency and green solvent was selected out of a series of solvents for extracting the phenolic compounds from DLT. TPC, TFC, ABTS^+•^, FRAP, and *α*-GIA derived from ChCl-LevA-based ultrasonic-assisted extraction were simultaneously optimized. Under optimum conditions (water content in ChCl-LevA reaching 45%, sonication temperature of 50 °C, and extraction time of 30 min), the experimentally measured results were consistent with the predicted results. High contents of (-)-epigallocatechin, catechin, protocatechuic acid, isoquercitrin and kaempferol 3-*O*-rutinoside were observed in ChCl-MaA-based UAE extracts. The contents of individual phenolics in ChCl-LevA-based UAE extracts were evidently higher than those in the other extracts, which indicates that ChCl-LevA is a high-efficient solvent for extracting the phenolic antioxidants from DLT. Based on the present results, ultrasonic-assisted extraction approach using ChCl-LevA as solvent can serve as a high-efficient eco-friendly method to extract natural bioactive phenolics from DLT. Moreover, ChCl-LevA could be used as a natural anti-oxidant and α-glucosidase inhibitor with health care effects.

## Figures and Tables

**Table 1:** Lists and physical-chemical properties of deep eutectic solvent (DES) prepared in this study.

No.	Component A	Component B	Component C	Abbreviations	Molar Ratio (mol/mol)	pH	Viscosity (mPa·s, 30% Water, 25 °C)
1	Choline chloride	Tetramethylene glycol	-	ChCl-TetG-1	1:1	3.15 ± 0.19	188 ± 1.38
2	Choline chloride	Tetramethylene glycol	-	ChCl-TetG-2	1:2	2.48 ± 0.09	122 ± 3.26
3	Choline chloride	Ethylene glycol	-	ChCl-EthG	1:1	4.26 ± 0.13	261 ± 6.87
4	Choline chloride	Triethylene glycol	-	ChCl-TriG	1:4	3.10 ± 0.08	96.35 ± 1.95
5	Choline chloride	1,2-Propanediol	-	ChCl-ProP	1:2	4.58 ± 0.05	173 ± 1.77
6	Choline chloride	Urea	-	ChCl-Urea	1:2	8.65 ± 0.05	127 ± 3.11
7	Choline chloride	Tartaric acid	-	ChCl-TarA	1:2	0.04 ± 0.02	173 ± 6.69
8	Choline chloride	Glycerol	-	ChCl-Gly	1:1	5.15 ± 0.12	205 ± 5.59
9	Choline chloride	Formic acid	-	ChCl-FA	1:1	0.85 ± 0.10	87.63 ± 2.18
10	Choline chloride	Lactic acid	-	ChCl-LA	1:1	0.81 ± 0.14	129 ± 4.95
11	Choline chloride	Levulinic acid	-	ChCl-LevA	1:2	1.36 ± 0.07	124 ± 2.16
12	Choline chloride	Malic acid	Xylitol	ChCl-MaA-Xyl	1:1:1	0.73 ± 0.04	143 ± 2.32
13	Choline chloride	Malic acid	Proline	ChCl-MaA-Pro	1:1:1	2.77 ± 0.17	233 ± 8.87
14	Betaine	Malic acid	Glucose	Bet-MaA-Glu	1:1:1	2.67 ± 0.13	120 ± 8.90
15	Betaine	Lactic acid	-	Bet-LA	1:2	3.09 ± 0.18	126 ± 3.18
16	Betaine	Glycerol	-	Bet-Gly	1:1	6.44 ± 0.18	242 ± 2.33
17	Betaine	Citric acid	-	Bet-CA	1:1	2.72 ± 0.14	295 ± 2.98

**Table 2:** Experimental and predicted results of the DLT extracts based on central composite design (CCD).

Run	Independent Variables			Responses									
	X1: Water Content (WC) (%)	B: t (min)	C: T (°C)	TPC (mg GAE/g DW)		TFC (mg RE/g DW)		ABTS^+^ (mmol TE/g DW)		FRAP (μmol Fe(II)E/g DW)		*α*-GIA (mmol AE/g DW)	
				Experimental Value (Exp.)	Predictive Value (Pred.)	Exp.	Pred.	Exp.	Pred.	Exp.	Pred.	Exp.	Pred.
1	30.00 (0)	3.18 (−1.68)	40.00 (0)	18.93 ± 2.10	31.03	26.41 ± 2.95	35.84	293 ± 6.94	350	5600 ± 145	5648	59.83 ± 2.29	53.67
2	30.00 (0)	20.00 (0)	40.00 (0)	91.34 ± 5.22	83.23	73.08 ± 3.42	72.79	484 ± 9.04	463	6038 ± 257	5842	173 ± 0.72	151
3	15.00 (−1)	10.00 (−1)	30.00 (−1)	37.09 ± 1.96	27.90	30.55 ± 1.89	26.92	420 ± 7.16	372	4691 ± 213	4568	61.50 ± 5.09	62.80
4	30.00 (0)	20.00 (0)	23.18 (−1.68)	36.98 ± 2.63	47.47	41.21 ± 1.13	41.52	437 ± 7.98	472	4570 ± 139	4959	143 ± 4.68	136
5	30.00 (0)	20.00	40.00 (0)	83.18 ± 2.02	83.23	65.61 ± 1.40	72.79	474 ± 6.43	463	5664 ± 31.91	5843	117 ± 2.06	151
6	15.00 (−1)	30.00 (+1)	30.00 (−1)	66.29 ± 3.69	61.64	40.15 ± 4.28	45.75	411 ± 1.29	401	4373 ± 77	4090	88.22 ± 0.70	84.46
7	15.00 (−1)	10.00 (−1)	50.00 (+1)	39.74 ± 1.55	33.75	42.28 ± 3.59	40.21	351 ± 16.28	323	4711± 154	4714	67.44 ± 6.82	62.69
8	45.00 (+1)	10.00 (−1)	30.00 (−1)	45.48 ± 2.97	37.15	32.28 ± 2.60	28.27	376 ± 12.01	352	5588 ± 129	5342	144 ± 5.24	154
9	30.00 (0)	20.00 (0)	40.00 (0)	82.84 ± 3.17	83.23	74.95 ± 1.62	72.79	491 ± 10.09	463	5865 ± 121	5843	157 ± 3.05	151
10	45.00 (+1)	30.00 (+1)	30.00 (−1)	91.00 ± 3.10	89.22	77.75 ± 0.28	76.96	488 ± 19.59	489	5817 ± 192	5823	151 ± 0.03	158
11	30.00 (0)	36.82 (+1.68)	40.00 (0)	81.92 ± 3.56	80.81	83.21 ± 0.40	77.82	461 ± 11.44	442	5773 ± 235	5712	124 ± 4.50	127
12	45.00 (+1)	10.00 (−1)	50.00 (+1)	73.87 ± 1.38	70.75	65.08 ± 3.42	56.63	453 ± 3.81	436	5632 ± 217	5924	141 ± 9.06	148
13	15.00 (−1)	30.00 (+1)	50.00 (+1)	40.31 ± 2.41	40.88	40.28 ± 3.46	41.44	298 ± 6.93	295	4056 ± 147	4310	152 ± 2.59	145
14	30.00 (0)	20.00 (0)	56.82 (+1.68)	57.78 ± 1.39	58.27	58.01 ± 3.21	61.74	450 ± 4.61	453	6034 ± 39.80	5633	177 ± 1.16	181
15	55.23 (+1.68)	20.00 (0)	40.00 (0)	91.11 ± 2.09	94.39	60.81 ± 3.72	66.86	432 ± 5.60	431	5954 ± 225	5848	220 ± 8.48	207
16	30.00 (0)	20.00 (0)	40.00 (0)	76.29 ± 1.50	83.23	79.61 ± 0.92	72.79	461 ± 11.14	463	6010 ± 121	5843	162 ± 0.34	151
17	30.00 (0)	20.00 (0)	40.00 (0)	86.75 ± 1.90	83.23	78.81 ± 2.87	72.79	413 ± 4.02	463	5921 ± 267	5843	137 ± 5.41	151
18	45.00 (+1)	30.00 (+1)	50.00 (+1)	94.79 ± 2.24	96.22	86.95 ± 3.40	87.72	495 ± 4.61	516	6348 ± 101	6479	211 ± 2.22	213
19	30.00 (0)	20.00 (0)	40.00 (0)	80.86 ± 0.49	83.23	65.35 ± 3.67	72.79	465 ± 9.22	463	5555 ± 36.18	5843	159 ± 0.92	151
20	4.77 (−1.68)	20.00 (0)	40.00 (0)	32.38 ± 2.61	40.09	28.81 ± 0.92	26.80	222 ± 3.89	262	32800 ± 6.96	3373	64.02 ± 0.19	73.74

**Table 3:** Analysis of the variance of the fitted second-order polynomial models and validation of the responses under the optimal conditions.

Term	Df	*F* Values (*p* Values)				
		TPC	TFC	ABTS^+•^	FRAP	*α*-GIA
Mode	9	17.06 (<0.0001) ***	16.18 (<0.0001) ***	6.91 (0.0028) **	30.10 (<0.0001) ***	16.21 (<0.0001) ***
*X*_1_	1	51.83 (<0.0001) ***	38.28 (<0.0001) ***	23.26 (<0.0001) ***	114.36 (<0.0001) ***	79.44 (<0.0001) ***
*X*_2_	1	43.55 (<0.0001) ***	42.06 (<0.0001) ***	6.95 (0.0249) *	0.48 (0.5151) ^ns^	23.67 (<0.0001) ***
*X*_3_	1	2.05 (0.1823) ^ns^	9.76 (0.0108) *	0.28 (0.6067) ^ns^	34.26 (0.0011) *	9.24 (0.0125) **
*X*_1 × 2_	1	2.45 (0.1489) ^ns^	8.82 (0.0141) *	3.88 (0.0773) ^ns^	14.71 (0.0086) **	0.55 (0.4761) ^ns^
*X*_1 × 3_	1	5.61 (0.0394) *	2.24 (0.1650) ^ns^	5.95 (0.0349) *	3.04 (0.1316) ^ns^	0.071 (0.7950) ^ns^
*X*_2 × 3_	1	5.15 (0.0466) *	3.06 (0.1107) ^ns^	1.09 (0.3201) ^ns^	0.089 (0.7761) ^ns^	6.77 (0.0264) *
*X*_1_^2^	1	6.70 (0.0270) *	23.99 (0.0006) ***	16.59 (0.0022) **	87.50 (< 0.0001) ***	0.74 (0.4087) ^ns^
*X*_2_^2^	1	19.56 (0.0013) **	9.07 (0.0131) *	5.57 (0.0400) *	1.52 (0.2631) ^ns^	24.71 (0.0006) ***
*X*_3_^2^	1	24.17 (0.0006) ***	15.94 (0.0025) **	1.12 (0.9786) ^ns^	17.22 (0.0061) **	0.37 (0.5573) ^ns^
Residual	10	686.94	505.88	14811.05	847400.00	2709.45
Lack of fit (F-value)	5	4.22	1.60	2.85	3.53	0.32
Lack of fit (*p*-value)	5	0.0798	0.3088	0.1372	0.0964	0.8819
*R* ^2^		0.9389	0.9701	0.9296	0.9849	0.9507
Adj *R*^2^		0.8838	0.9054	0.8778	0.9522	0.9438
Pre *R*^2^		0.8087	0.9763	0.8014	0.9716	0.8119
Adequacy precision		11.657	10.900	9.321	20.811	10.304
Validation of the optimal UAE conditions						
Predicted results		96.21	86.58	481	6473	218
Experimental results		91.38 ± 7.20	84.12 ± 3.47	492 ± 7.33	6235 ± 121	230 ± 7.59
Error (%)		5.02	2.84	2.12	3.68	5.47
*p* value (Predicted vs. Experimental)		0.78	1.02	0.27	0.34	1.07

ns, not significant (*p* > 0.05). *, **, and ***, significant at *p* < 0.05, *p* < 0.01, and *p* < 0.001, respectively.

**Table 4:** Individual phenolic contents in the DLT extracts obtained by different extraction methods.

Individual Phenolic Content (μg/g DW)	Water-Based HE	MeOH-Based HE	ChCl-LevA-Based HE	ChCl-LevA-Based MWE	ChCl-LevA-Based UAE
Gallic acid	463 ± 5.74 ^e^	596 ± 3.43 ^d^	1009± 18.41 ^a^	924 ± 50.14 ^b^	829 ± 15.63 ^c^
(-)-Epigallocatechin	2781 ± 19.57 ^d^	1503 ± 33.26 ^e^	3003 ± 39.77 ^c^	4050 ± 19.13 ^b^	4272 ± 84.86 ^a^
Catechin	0.00	0.00	4338 ± 28.75 ^c^	5025 ± 106 ^b^	5268 ± 24.53 ^a^
Protocatechuic acid	15.06 ± 1.92 ^e^	284 ± 7.96 ^d^	418 ± 32.22 ^c^	575 ± 4.36 ^b^	644 ± 1.65 ^a^
Vanillic acid	565 ± 6.42	0.00	0.00	0.00	0.00
Rutin	128 ± 8.62 ^c^	119 ± 2.33 ^c^	169 ± 1.62 ^b^	255 ± 5.10 ^a^	239 ± 26.88 ^a^
Isoquercitrin	2126 ± 10.32 ^a^	2900 ± 63.22 ^b^	2031 ± 24.12 ^a^	3411 ± 12.93 ^c^	3500 ± 86.07 ^d^
Kaempferol 3-*O*-rutinoside	2994 ± 71.44 ^c^	1550 ± 38.72 ^e^	2026 ± 29.01 ^d^	3669 ± 25.42 ^b^	3717 ± 97.71 ^a^
Myricetin	791 ± 11.37 ^a^	584 ± 10.00 ^c^	530 ± 33.49 ^c^	711 ± 2.16 ^b^	716 ± 11.85 ^b^
Phloretin	24.65 ± 3.45 ^b^	0.00	18.85 ± 0.39 ^c^	29.57 ± 0.29 ^a^	31.33 ± 0.51 ^a^

Water-based HE, heating extraction with water; MeOH-based HE, heating extraction with MeOH; ChCl-LevA-based HE, heating extraction with ChCl-LevA; ChCl-LevA-based MWE, microwave extraction with ChCl-LevA; ChCl-LevA-based UAE, ultrasonic-assisted extraction with ChCl-LevA. Different lowercase letters (a–e) in same row indicate significant difference (*p* < 0.05).

## Data Availability

Not applicable.

## Supplementary Materials

The following are available online at https://www.mdpi.com/article/10.3390/foods10112527/s1, Figure S1: Pareto chart of the standardized effects on the TPC (A) and TFC (B) by Full factorial design experiments. Water content in DES (A), liquid to solid ratio (B), extraction time (C), ultrasonication power (D), and sonication temperature (E); Figure S2: Contour plots of interaction effects between the independent variables on TPC (A-B), TFC (C), ABTS^+•^ (D), FRAP (E) and *α*-GIA (F) of the DLT extracts. Table S1: Experimental design and results of full factorial design experimental; Table S2: Independent process variables with experimental ranges and levels of response surface methodology.
