# Supplementary material for: Customized Deep Eutectic Solvents as Green Extractants for Ultrasonic-Assisted Enhanced Extraction of Phenolic Antioxidants from Dogbane Leaf-Tea
Source: Foods. 2021 Oct 21;10(11):2527. doi: 10.3390/foods10112527 (PMC8620010; doi:10.3390/foods10112527)
Supplement: Supplementary file 1 [file foods-10-02527-s001.zip › Table S1.pdf]

Table S1

Experimental design and results of full factorial design experimental

Run	Independent variables					Responses	
	A: WC (%)	B: L/S (mL/g)	C: t (min)	D: Power (W)	E: T (°C)	Y1: TPC (mg GAE/g DW)	Y2: TFC (mg RE/g DW)
1	15.00 (-1)	10.00 (-1)	20.00 (+1)	100.00 (-1)	35.00 (-1)	63.86 ± 6.97	43.68 ± 2.25
2	45.00 (+1)	10.00 (-1)	5.00 (-1)	100.00 (-1)	35.00 (-1)	66.06 ± 1.97	51.85 ± 4.51
3	15.00 (-1)	30.00 (+1)	20.00 (+1)	100.00 (-1)	50.00 (+1)	70.50 ± 5.64	55.62 ± 5.92
4	15.00 (-1)	10.00 (-1)	5.00 (-1)	100.00 (-1)	50.00 (+1)	73.92 ± 4.55	35.22 ± 2.73
5	45.00 (+1)	10.00 (-1)	5.00 (-1)	200.00 (+1)	50.00 (+1)	78.14 ± 1.77	34.42 ± 0.31
6	45.00 (+1)	30.00 (+1)	5.00 (-1)	100.00 (-1)	50.00 (+1)	73.36 ± 2.53	55.99 ± 3.74
7	45.00 (+1)	10.00 (-1)	20.00 (+1)	200.00 (+1)	35.00 (-1)	82.13 ± 1.05	63.88 ± 0.12
8	45.00 (+1)	30.00 (+1)	20.00 (+1)	100.00 (-1)	35.00 (-1)	80.14 ± 5.28	60.35 ± 6.23
9	15.00 (-1)	30.00 (+1)	5.00 (-1)	200.00 (+1)	50.00 (+1)	67.46 ± 3.79	35.09 ± 2.83
10	15.00 (-1)	30.00 (+1)	20.00 (+1)	200.00 (+1)	35.00 (-1)	63.36 ± 3.76	45.42 ± 6.91
11	15.00 (-1)	10.00 (-1)	20.00 (+1)	200.00 (+1)	50.00 (+1)	78.86 ± 5.97	48.80 ± 4.88
12	15.00 (-1)	10.00 (-1)	5.00 (-1)	200.00 (+1)	35.00 (-1)	55.58 ± 5.21	32.68 ± 1.02
13	45.00 (+1)	10.00 (-1)	20.00 (+1)	100.00 (-1)	50.00 (+1)	82.55 ± 4.49	66.27 ± 3.21
14	45.00 (+1)	30.00 (+1)	5.00 (-1)	200.00 (+1)	35.00 (-1)	78.56 ± 5.11	55.62 ± 1.24
15	45.00 (+1)	30.00 (+1)	20.00 (+1)	200.00 (+1)	50.00 (+1)	84.79 ± 0.51	84.62 ± 2.77
16	15.00 (-1)	30.00 (+1)	5.00 (-1)	100.00 (-1)	35.00 (-1)	56.75 ± 3.44	41.35 ± 4.02
