# Supplementary material for: Customized Deep Eutectic Solvents as Green Extractants for Ultrasonic-Assisted Enhanced Extraction of Phenolic Antioxidants from Dogbane Leaf-Tea
Source: Foods. 2021 Oct 21;10(11):2527. doi: 10.3390/foods10112527 (PMC8620010; doi:10.3390/foods10112527)
Supplement: Supplementary file 1 [file foods-10-02527-s001.zip › Table S2.pdf]

Table S2

Independent process variables with experimental ranges and levels of response surface methodology.

Independent Variable	Code				
	-1.68 (-α)	-1	0	+1	+1.68 (+α)
X_1: Water content (WC, %)	4.77	15	30	45	55.23
X_2: Ultrasonic time (t, min)	3.18	10	20	30	36.82
X_3: Ultrasonic temperature (T, °C)	23.18	30	40	50	56.82
