# Supplementary figures and images for: Customized Deep Eutectic Solvents as Green Extractants for Ultrasonic-Assisted Enhanced Extraction of Phenolic Antioxidants from Dogbane Leaf-Tea
Source: Foods. 2021 Oct 21;10(11):2527. doi: 10.3390/foods10112527 (PMC8620010; doi:10.3390/foods10112527)

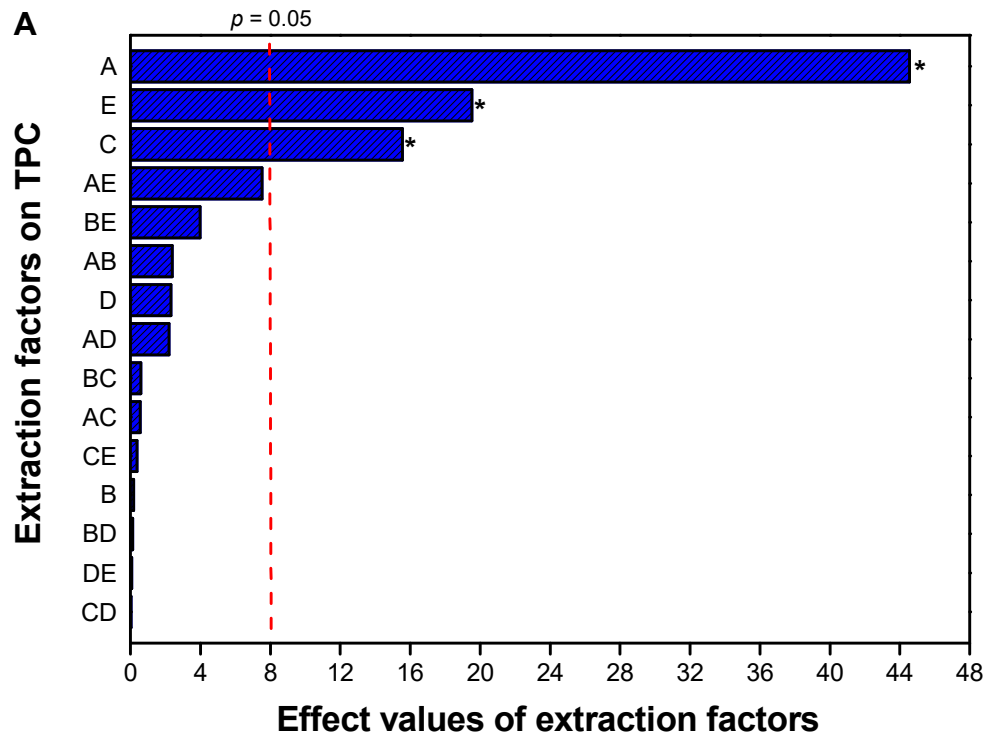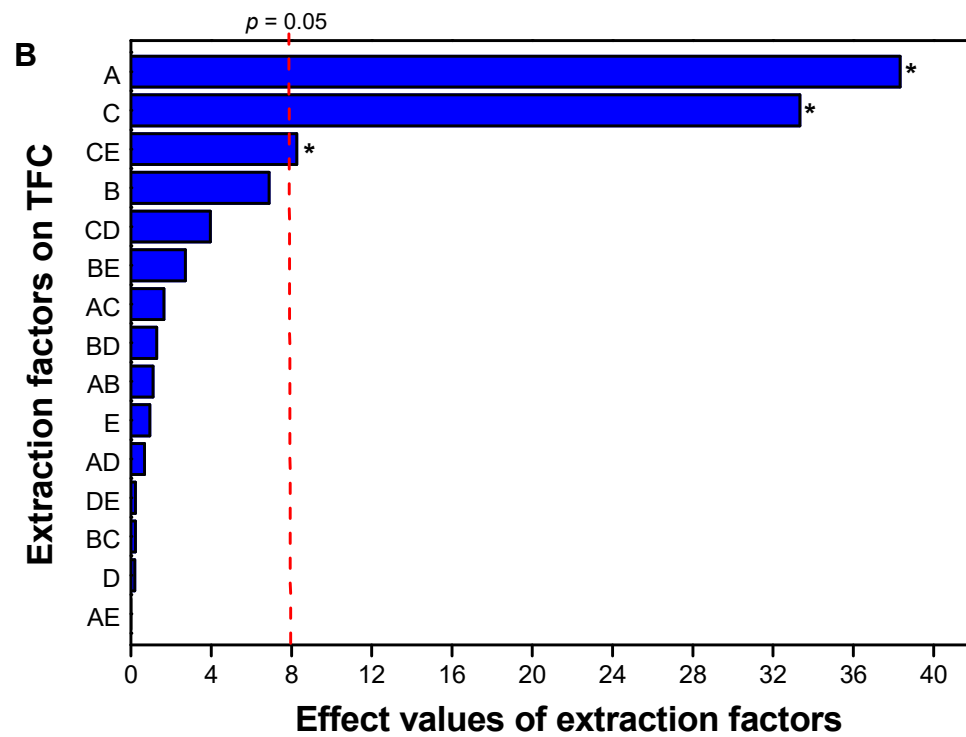

Supplement: Supplementary file 1 [file foods-10-02527-s001.zip › Figure S1.pdf]
